# Normal colonic mucosa in hereditary non-polyposis colorectal cancer shows no generalised increase in somatic mutation.

**DOI:** 10.1038/bjc.1995.208

**Published:** 1995-05

**Authors:** G. T. Williams, J. M. Geraghty, F. Campbell, M. A. Appleton, E. D. Williams

**Affiliations:** Department of Pathology, University of Wales College of Medicine, Heath Park, Cardiff, UK.

## Abstract

Hereditary non-polyposis colorectal cancer (HNPCC) has recently been linked to germline defects of DNA repair genes. Colorectal tumours in HNPCC frequently show DNA microsatellite instability, but it is not certain whether this mutator phenotype occurs throughout the morphologically normal colonic mucosa. We have previously used the mPAS histochemical technique in human colorectal mucosa to identify a polymorphism for O-acetyltransferase activity that shows monogenic inheritance and to show that crypt-restricted loss of O-acetyltransferase activity in heterozygotes is due to somatic mutation. We have now used this histochemical technique to measure the somatic mutation frequency in the uninvolved colon of 12 heterozygous patients with HNPCC, 15 with ileocaecal Crohn's disease and 16 with sporadic colorectal cancer (CRC). HNPCC patients showed a significant increase in mutation frequency with age (Mann-Whitney U, P = 0.02). In HNPCC patients aged < 49 years the mean stem cell mutation frequency was significantly lower than in the slightly younger group of patients with Crohn's disease (0.8 +/- 0.9 x 10(-4) vs 3.5 +/- 3.3 x 10(-4), P < 0.01), probably reflecting an increased mutation rate relating to chronic mucosal damage in Crohn's disease. Although not statistically significant, the stem cell mutation frequency was slightly less in HNPCC patients > 50 years than in sporadic CRC cases (4.9 +/- 3.4 x 10(-4) vs 5.9 +/- 3.6 x 10(-4), P > 0.5). We conclude that germline defects in HNPCC do not result in a generalised increase in liability to mutation in normal colonic mucosa but that a second, somatic, event is required. We postulate that this second event occurs in crypt stem cells at low frequency, giving rise to scattered individual crypts composed of mutation-prone cells. The cells in these crypts are then at high risk of acquiring the mutations that lead to adenomas, and to rapid progression to carcinoma.


British Journal of Cancer (1995) 71, 1077-1080

© 1995 Stockton Press All rights reserved 0007-0920/95 $12.00

### Normal colonic mucosa in hereditary non-polyposis colorectal cancer shows no generalised increase in somatic mutation

GT Williams[1], JM Geraghty[2], F Campbell[1], MAC Appleton[1] and ED Williams[2]

[1]*Departments of Pathology, University of Wales College of Medicine, Heath Park, Cardiff, CF4 4XN, UK;* [2]*Addenbrooke's Hospital, Hills Road, Cambridge CB2 2QQ, UK.*

**Summary** Hereditary non-polyposis colorectal cancer (HNPCC) has recently been linked to germline defects of DNA repair genes. Colorectal tumours in HNPCC frequently show DNA microsatellite instability, but it is not certain whether this mutator phenotype occurs throughout the morphologically normal colonic mucosa. We have previously used the mPAS histochemical technique in human colorectal mucosa to identify a polymorphism for *O*-acetyltransferase activity that shows monogenic inheritance and to show that crypt-restricted loss of *O*-acetyltransferase activity in heterozygotes is due to somatic mutation. We have now used this histochemical technique to measure the somatic mutation frequency in the uninvolved colon of 12 heterozygous patients with HNPCC, 15 with ileocaecal Crohn's disease and 16 with sporadic colorectal cancer (CRC). HNPCC patients showed a significant increase in mutation frequency with age (Mann–Whitney *U*, $P=0.02$). In HNPCC patients aged <49 years the mean stem cell mutation frequency was significantly lower than in the slightly younger group of patients with Crohn's disease ($0.8 \pm 0.9 \times 10^{-4}$ *vs* $3.5 \pm 3.3 \times 10^{-4}$, $P<0.01$), probably reflecting an increased mutation rate relating to chronic mucosal damage in Crohn's disease. Although not statistically significant, the stem cell mutation frequency was slightly less in HNPCC patients >50 years than in sporadic CRC cases ($4.9 \pm 3.4 \times 10^{-4}$ *vs* $5.9 \pm 3.6 \times 10^{-4}$, $P>0.5$). We conclude that germline defects in HNPCC do not result in a generalised increase in liability to mutation in normal colonic mucosa but that a second, somatic, event is required. We postulate that this second event occurs in crypt stem cells at low frequency, giving rise to scattered individual crypts composed of mutation-prone cells. The cells in these crypts are then at high risk of acquiring the mutations that lead to adenomas, and to rapid progression to carcinoma.

**Keywords:** somatic mutation; HNPCC; colorectal cancer; Crohn's disease

Colorectal carcinoma (CRC) is known to occur as a familial tumour as part of two distinct syndromes, familial adenomatous polyposis (FAP) and hereditary non-polyposis colorectal cancer (HNPCC). The gene for FAP (*APC*) has been identified on chromosome 5q21 (Kinzler *et al.*, 1991), and inheritance of a mutated allele is associated with the development during the second or third decade of life of very many colorectal adenomas, a small number of which are highly likely to progress to adenocarcinoma in early to mid adult life. HNPCC appears to be a more genetically heterogeneous condition, in that inherited abnormalities of at least four genes on chromosomes 2p (Peltomäki *et al.*, 1993), 2q (Nicolaides *et al.*, 1994), 3p (Lindblom *et al.*, 1993) and 7p (Nicolaides *et al.*, 1994) appear to be involved in different kindreds. CRC in HNPCC usually presents later in life than in FAP, and is more often right-sided, more frequently diploid or near-diploid and possibly more often of mucinous or signet ring cell type than sporadic CRC (Lynch *et al.*, 1993). While multiple carcinomas may occur, the background bowel rarely contains more than a few adenomas, although these are often larger and are claimed to be more prone to malignant progression than their sporadic counterparts (Jass and Stewart, 1992). Extracolonic tumours occur in both FAP and HNPCC patients, notably adenomas and carcinomas of the more proximal gastrointestinal tract, desmoid tumours, thyroid, brain and liver tumours in FAP (Phillips *et al.*, 1994) and endometrial, gastric, ovarian and urinary tract carcinomas in HNPCC (Lynch *et al.*, 1993).

The *APC* gene is thought to act as a tumour-suppressor gene and, although the function of its product is not established, there is evidence to suggest that it is involved in cell adhesion (Su *et al.*, 1993). The four putative HNPCC genes so far identified, on the other hand, appear to be involved in DNA repair. Colorectal adenomas and carcinomas, and extracolonic carcinomas, of HNPCC patients show a high frequency of DNA microsatellite instability (Aaltonen *et al.*, 1994), and human homologues of the prokaryotic DNA mismatch repair genes *mutS* and *mutL* map to the regions of chromosomes 2, 3 and 7 that have been shown by linkage studies to bear the HNPCC loci. Moreover, germline mutations of these repair genes occur in affected patients (Fishel *et al.*, 1993; Leach *et al.*, 1993; Bronner *et al.*, 1994; Nicolaides *et al.*, 1994). HNPCC patients might therefore be expected to show an increased frequency of somatic mutation in a wide range of other genes. Loss of heterozygosity for chromosomes 2 or 3 is not common in HNPCC tumours (Aaltonen *et al.*, 1993; Lindblom *et al.*, 1993) and it is not certain whether the gene is acting dominantly, conferring increased mutation in the normal colon as well as in tumours, or whether its action is confined to tumours. Evidence suggesting the latter is derived from the fact that a lymphoblastoid cell line from an HNPCC-affected individual was proficient in mismatch repair (Parsons *et al.*, 1993) and that normal tissue (of unspecified type) from HNPCC patients was not found to show microsatellite instability (Aaltonen *et al.*, 1993). However, the possibility that the inherited DNA defect is confined to tissues with an increased incidence of cancer, e.g. colonic mucosa, is not excluded. We have therefore set out to determine whether the somatic mutation rate is increased in the morphologically normal-appearing colon of HNPCC patients.

We have shown that a polymorphism exists in human colon for the *O*-acetylation of sialic acid in mucus glycoproteins which can be demonstrated in tissue sections using mPAS histochemistry (Fuller *et al.*, 1990; Campbell *et al.*, 1994*a*). This technique, which is a modification of the periodic-acid Schiff procedure, allows non-*O*-acetylated sialomucins to be distinguished from *O*-acetylated sialomucins (Veh *et al.*, 1992). In the UK approximately 10% of the adult population show a phenotype resulting from low or absent mucus glycoprotein *O*-acetylation, while about 90% show high *O*-acetylation. Approximately one-half of adults with the high-activity phenotype show infrequent scat-

Correspondence: GT Williams
Received 3 October 1994; revised 14 November 1994; accepted 19 December 1994

tered discordant low-activity crypts. The hypothesis that these discordant crypts are the result of somatic mutation in crypt stem cells in heterozygous individuals has been verified by showing that they are lacking in children, and that they increase in frequency after exposure to a mutagen (Fuller *et al*., 1990; Campbell *et al*., 1994*b*). Although the gene involved has not been identified, a monogenic basis for the polymorphism has been confirmed by showing that the phenotype frequency varies between Sino-Japanese and other races, but that in all populations the phenotype frequency is close to that predicted by the Hardy–Weinberg law (Campbell *et al*., 1994*a*). Most discordant crypts in adults are wholly involved and are considered to represent stem cell mutations which accumulate with time. A minority show partial involvement; those from observations in man and animals have been shown to represent recent mutations (Campbell *et al*., 1994*b*). We have therefore quantified the frequency of discordant crypts in microscopically normal colon from patients with HNPCC, and compared this with their frequency in uninvolved colon from patients with either Crohn's disease (for younger HNPCC patients) or sporadic right-sided CRC (for older HNPCC patients). These two groups were chosen for the comparison because of the unavailability of an adequate number of right-sided colonic resection specimens from normal individuals with a wide age range.

### Materials and methods

Twenty six HNPCC patients who had undergone large bowel resection for CRC were identified from registers maintained in Cambridge and Cardiff. They were derived from a total of 19 families, and all satisfied the Amsterdam criteria for a diagnosis of HNPCC (Vasen *et al*., 1991) with (a) three or more relatives with histologically verified CRC, one of whom is a first-degree relative of the other two (b) CRC involving at least two generations and (c) one or more CRCs diagnosed before the age of 50 years. In 19 cases the CRC occurred proximal to the splenic flexure. None of the patients had been treated with radiotherapy or chemotherapy. Paraffin blocks from these HNPCC specimens, from 32 right hemicolectomy specimens for ileocaecal Crohn's disease and from 40 consecutive hemicolectomy specimens for sporadic primary right-sided CRC collected prospectively were retrieved from departmental files. None of these patients had received radiotherapy or cancer chemotherapy, but two of the Crohn's disease patients had received the mutagen azathioprine for immunosuppression.

Blocks of uninvolved microscopically normal colonic resection margin mucosa were selected and 5 μm step sections, cut at 80 μm intervals apart, were stained by the mPAS reaction (Veh *et al*., 1982). In this technique the oxidation step of a standard periodic acid–Schiff reaction is modified (using 1 mM sodium periodate at 4°C for 10 min) such that non-*O*-acetylated sialomucins are oxidised and subsequently stained magenta by the Schiff reagent while *O*-acetylated sialomucins are not. The total number of crypt profiles in each patient's sample was determined by counting the number of crypt profiles in the central step section manually using a hand-held tally and multiplying this by the number of step sections studied. At least 10 000 crypt profiles were counted in each case (means 13 898 for HNPCC, 14 215 for Crohn's disease, 20 135 for sporadic right-sided CRC) as samples of this size are necessary for reliable phenotyping and meaningful interpretation of results (Campbell *et al*., 1994*a*, *b*). For each case the phenotype was determined and in those exhibiting the heterozygous (high activity with scattered discordant low-activity crypts) phenotype the frequency of discordant crypts recorded. Each discordant crypt was categorised as being wholly involved when all mucin-containing cells in the profile showed the low-activity phenotype or partially involved when both phenotypes were present. The distribution of the three phenotypes in each of the three groups studied was compared with that predicted by the Hardy–Weinberg law (Vogel and Motulsky, 1986) using the $\chi^2$ test as described previously (Campbell *et al*., 1994*a*) and the frequencies of discordant (mutated) crypts in each group were compared using the Mann–Whitney *U*-test.

### Results

Table I shows the age, sex and mPAS phenotype distributions in the three patient groups studied. As would be expected, patients undergoing right hemicolectomy for Crohn's disease were significantly younger than those with sporadic CRC, while patients with HNPCC had a broad age range. The frequency distribution of the three mPAS phenotypes was similar in all three patient groups, and showed no significant difference ($\chi^2$, $P > 0.9$) from that predicted by the Hardy–Weinberg law for a single polymorphic gene.

Table II shows the frequencies of discordant (mutated) crypts in patients with scattered mPAS-positive crypts in a predominantly mPAS-negative background, i.e. those patients considered to be heterozygous for *O*-acetyltransferase activity and therefore informative for measuring the frequency of somatic mutation. Since HNPCC patients showed a much wider age range than the other two groups, they have also been divided into those aged $<49$ years and those $>50$ years. This is particularly important for the comparison of wholly mutated crypt frequencies because they result from fixed stem cell mutations and reflect the lifetime accumulated mutational load (Campbell *et al*., 1994*b*).

In informative HNPCC patients the total frequency of mutated crypts ranged from $0.7 \times 10^{-4}$ to $12.7 \times 10^{-4}$. The mean frequency in the five patients aged 28–48 years was $1.5 \pm 0.8 \times 10^{-4}$ (median $1.0 \times 10^{-4}$), and in the seven patients aged 51–80 years it was $6.3 \pm 4.3 \times 10^{-4}$ (median $4.3 \times 10^{-4}$), a significant difference (Mann–Whitney *U*, $P = 0.02$) that was largely accounted for by a difference in the frequencies of wholly involved crypts ($P = 0.01$). The frequency of partially involved crypts was not significantly different in the two age groups.

**Table I** mPAS phenotype distributions in the three patient groups studied

| | *Number of cases* | *M:F ratio* | *Median age (range)* | *Uniform mPAS positive* | *mPas Phenotype* — *Uniform mPAS negative* observed | *Uniform mPAS negative* predicted[a] | *mPAS negative with discordant crypts* observed | *mPAS negative with discordant crypts* predicted[a] |
|---|---|---|---|---|---|---|---|---|
| HNPCC | 26 | 1.0:1.0 | 47 (28–80) | 2 | 12 | 13 | 12 | 11 |
| Crohn's disease | 32 | 1.0:2.0 | 30 (19–53) | 2 | 15 | 18 | 15 | 12 |
| Right-sided CRC | 40 | 1.0:1.5 | 72 (47–94) | 5 | 19 | 17 | 16 | 18 |

[a]Predictions according to the Hardy–Weinberg law (Vogel and Motulsky, 1986) based on the frequency of mPAS-positive phenotypes.

**Table II** Frequencies of discordant (mutated) crypts in informative patients (presumed heterozygous for *O*-acetyltransferase activity)

| | *Number of informative cases* | *Median age (range)* | *Mean frequency of mutated crypts x $10^{-4}$ (median)* Wholly involved | Partially involved | Total |
|---|---|---|---|---|---|
| HNPCC (all) | 12 | 52 (28–80) | 3.2 ± 3.4** (1.7) | 1.1 ± 1.3 (1.0) | 4.3 ± 4.1 (3.0) |
| HNPCC <49 years | 5 | 32 (28–48) | 0.8 ± 0.9*‡ (0.7) | 0.7 ± 0.7 (0.9) | 1.5 ± 0.8† (1.0) |
| HNPCC >50 years | 7 | 56 (51–80) | 4.9 ± 3.4* (3.6) | 1.4 ± 1.6 (1.0) | 6.3 ± 4.3† (4.3) |
| Crohn's disease | 15 | 27 (19–46) | 3.5 ± 3.3‡ (2.4) | 0.7 ± 1.4 (0) | 4.2 ± 4.0 (3.1) |
| Right-sided CRC | 16 | 75 (62–94) | 5.9 ± 3.6** (5.5) | 0.6 ± 0.6 (0.5) | 6.6 ± 4.0 (6.1) |

Statistically significant differences (Mann–Whitney *U*) between groups are: * $P = 0.01$ for wholly involved crypts, young *vs* old HNPCC patients. **$P < 0.05$ for wholly involved crypts, all HNPCC patients *vs* right-sided CRC. †$P = 0.02$ for all (wholly + partially) involved crypts, young *vs* old HNPCC patients. ‡$P < 0.01$ for wholly involved crypts, young HNPCC *vs* Crohn's disease.

Informative patients with Crohn's disease were aged 19–46 years (median 27 years) and had total mutated crypt frequencies ranging from $0.9 \times 10^{-4}$ to $15.5 \times 10^{-4}$ with a mean of $4.2 \pm 4.0 \times 10^{-4}$ and a median of $3.1 \times 10^{-4}$. However the two highest frequencies ($15.5 \times 10^{-4}$ in a 36-year old and $8.0 \times 10^{-4}$ in a 26-year old) were found in the two patients who had received preoperative azathioprine treatment. Comparison of the discordant crypt frequencies in all 15 informative Crohn's disease patients with the similarly aged younger group of five HNPCC cases shows a significantly higher frequency of wholly involved crypts in the Crohn's disease group ($P < 0.01$), but not of partially involved crypts ($P > 0.3$). Even when the two azathioprine-treated patients are excluded the difference for wholly involved crypts remains significant ($P < 0.02$).

The 16 informative patients with sporadic right-sided CRC were aged 62–94 years (median 75 years). Their mean total mutated crypt frequency was $6.6 \pm 4.0 \times 10^{-4}$ (median $6.1 \times 10^{-4}$), ranging from 0.5 to $12.0 \times 10^{-4}$. Comparison of the discordant crypt frequencies in these cases with all 12 HNPCC cases showed a significantly increased frequency of wholly mutated crypts in those with sporadic CRC ($P < 0.05$). However, this difference can be attributed to the different ages of the patients in the two groups. When the comparison is confined to the seven HNPCC patients aged >50 years (median 56 years) the statistical significance of the difference disappears ($P = 0.5$). No significant difference was found in the frequencies of partially involved discordant crypts between HNPCC and sporadic CRC patients.

### Discussion

The main finding in this study is that the somatic mutation frequency in the non-tumorous background colonic mucosa of patients with HNPCC, as assessed by mPAS histochemistry, is not significantly raised when compared with age-matched patients with sporadic right-sided CRC, and is significantly lower than that found in age-matched patients with Crohn's disease when assessed by the frequency of accumulated stem cell mutations (i.e. wholly involved discordant crypts). Our findings therefore support those of molecular studies in other tissues derived from HNPCC patients, and suggest that there is no generalised tissue-specific mutator phenotype in the colonic epithelium, the cell lineage in which most tumours arise in this inherited cancer syndrome. However, we cannot exclude the possibility that the gene responsible for *O*-acetyltransferase activity is mutated by a different mechanism from that relevant to HNPCC. The fact that the colonic mutation frequency in the young (<49 years) group of HNPCC patients was lower than in age-matched Crohn's disease patients suggests that it may represent the 'normal' cumulative mutation frequency at this age. While it is possible that the sporadic CRC group contains unrecognised HNPCC cases, we believe that this is unlikely to have had a major influence on the findings because the age distribution and frequency of somatic mutation in our patients with right-sided CRC were not significantly different from a previously reported group of rectal cancer patients (Campbell *et al.*, 1994*b*).

Since germline defects of the various genes resulting in HNPCC syndromes do not by themselves lead to a detectable generalised increased mutation frequency in the colon, a second event is needed to trigger the cascade of events leading to carcinogenesis. This is presumably a mutation in another gene which, together with the inherited mutation, allows expression of the increased liability to mutation. The nature of the second mutation can only be a matter for speculation at present. The simple explanation, that it is a somatically acquired defect in the second allele leading to loss of function of the HNPCC gene, has been questioned because loss of heterozygosity (LOH) of linked markers for the relevant chromosome loci is not a frequent finding in HNPCC tumours (Aaltonen *et al.*, 1993; Lindblom *et al.*, 1993). However, somatic point mutation of the second (wild-type) allele has been described in two CRCs in HNPCC patients with germline mutations of either *mut*L or *mut*S homologues (Leach *et al.*, 1993; Nicolaides *et al.*, 1994), indicating that this could still be an important route for acquiring the mutator phenotype. Another possibility is that liability to increased mutation requires somatic mutation of a second gene for its expression (Leach *et al.*, 1993). If the presumptive second mutation were to occur at the same frequency as the mutation we have observed in this study, this would imply that by the age of 50 years only some three or four crypts in every 10 000 would have acquired an increased somatic mutation rate. This would not be a sufficiently common event to be detectable by current methods of estimating somatic mutation rates *in vivo*. However, it could well be sufficient to result in the increased frequency of CRC in HNPCC patients, particularly when it is remembered that an accelerated mutation rate is likely to increase the chance of mutation in other DNA repair genes with a cascade effect.

The finding of an increased somatic mutation frequency in the colons of Crohn's disease patients is of interest, and is probably related to increased regenerative epithelial proliferation in chronic colitis. It is likely to be related to the known increased risk of colorectal cancer in chronic inflammatory bowel disease, both Crohn's colitis and ulcerative colitis (Gillen *et al.*, 1994). The fact that the highest mutation frequencies in Crohn's disease patients occurred in the two who had received azathioprine would be predicted from treatment with a known mutagen, and raises concerns that azathioprine therapy may add to the carcinogenic risk in inflammatory bowel disease. A recent retrospective study of long-term neoplasia risk after azathioprine treatment in patients with Crohn's disease and ulcerative colitis (Connell *et al.*, 1994) found a significant 2.5 fold increase in all neoplasms after more than 5 years of azathioprine, and a 17-fold increase in CRC, although this did not reach statistical significance (the absolute number of tumours was small). The same study compared the frequency of CRC in azathioprine-treated ulcerative colitis patients with matched patients who had not received the drug. Only a small non-significant increase was observed in the treated group, but this included an unspecified proportion of patients with short treatment times. Further studies of the risks of long-term azathioprine therapy are needed.

Our observations can be correlated with the clinical findings in FAP and HNPCC. The high frequency of colonic adenomas in FAP is consistent with a single somatic mutation of the normal allele of the *APC* gene leading to adenoma formation in these patients. These adenomas individually have a relatively low risk of progression to malignancy but they are so numerous that malignancy is virtually certain to occur in one or more polyps during the lifetime of an FAP patient. The infrequency of adenomas in HNPCC patients suggests that a single somatic mutation is not sufficient, but that at least two events are needed for adenoma formation. Our findings show that there is no general increase in somatic mutation, and are compatible with a sequence of events in which a single somatic mutation leads to an increased propensity to somatic mutation in the involved crypt only. A significant proportion of these 'mutation-prone' crypts then acquire the further events needed to give rise to an adenoma, also mutation prone, and with a high chance of progression to malignancy. This hypothesis is supported by the high frequency of microsatellite instability in both adenomas and carcinomas in HNPCC (Aaltonen *et al.*, 1994), and by the suggestion that adenomas in these patients are more prone to malignant progression than sporadic adenomas or those of FAP (Jass and Stewart, 1992).

**Acknowledgments**

We wish to thank Dr Fraser Campbell for statistical analyses, Dr J Sampson and Mrs A Williams from the Institute of Medical Genetics, Cardiff, Dr ER Maher and Mrs J Koch from the Department of Clinical Genetics, Cambridge, and the following pathologists: Dr HK Al-Rufaie (Bury St. Edmonds), Dr BJ Charnley (Merthyr Tydfil), Dr JS Dinnen (Hereford), Dr D Eakins (King's Lynn), Dr RW Fortt (Newport), Dr RJ Kellett (Abergavenny), and Dr NA Shepherd (Gloucester).

**References**

AALTONEN LA, PELTOMÄKI P, LEACH FS, SISTONEN P, PYLKKÄNEN L, MECKLIN J-P, JÄRVINEN H, POWELL SM, JEN J, HAMILTON SR, PETERSEN GM, KINZLER KW, VOGELSTEIN B AND DE LA CHAPELLE A. (1993). Clues to the pathogenesis of familial colorectal cancer. *Science*, **260**, 812-816.

AALTONEN LA, PELTOMÄKI P, MECKLIN JP, JÄRVINEN H, JASS JR, GREEN JS, LYNCH HT, WATSON P, TALLQVIST G, JUHOLA M, SISTONEN P, HAMILTON SR, KINZLER KW, VOGELSTEIN B AND DE LA CHAPELLE A. (1994). Replication errors in benign and malignant tumours from hereditary nonpolyposis colorectal cancer patients. *Cancer Res.*, **54**, 1645-1648.

BRONNER CE, BAKER SM, MORRISON PT, WARREN G, SMITH LG, LESCOE MK, KANE M, EARABINO C, LIPFORD J, LINDBLOM A, TANNERGÅRD P, BOLLAG RJ, GODWIN AR, WARD DC, NORDENSKJØLD M, FISHEL R, KOLODNER R AND LISKAY RM. (1994). Mutation in the DNA mismatch repair gene homologue *hMLH1* is associated with hereditary non-polyposis colon cancer. *Nature*, **368**, 258-261.

CAMPBELL F, APPLETON MAC, FULLER CE, GREEFF MP, HALLGRIMSSON J, KATOH R, NG OLI, SATIR A, WILLIAMS GT AND WILLIAMS ED. (1994*a*). Racial variation in the O-acetylation phenotype of human colonic mucosa. *J. Pathol.*, **174**, 169-174.

CAMPBELL F, FULLER CE, WILLIAMS GT AND WILLIAMS ED. (1994*b*). Human colonic stem cell mutation frequency with and without irradiation. *J. Pathol.*, **174**, 175-182.

CONNELL WR, KAMM MA, DICKSON M, BALKWILL AM, RITCHIE JK AND LENNARD-JONES JE. (1994). Long term neoplasia risk after azathioprine treatment in inflammatory bowel disease. *Lancet*, **343**, 1249-1252.

FISHEL R, LESCOE MK, RAO MRS, COPELAND NG, JENKINS NA, GARBER J, KANE M AND KOLODNER R. (1993). The human mutator gene homolog *MSH2* and its association with hereditary nonpolyposis colon cancer. *Cell*, **75**, 1027-1038.

FULLER CE, DAVIES RP, WILLIAMS GT AND WILLIAMS ED. (1990). Crypt restricted heterogeneity of goblet cell mucus glycoprotein in histologically normal human colonic mucosa: a potential marker of somatic mutation. *Br. J. Cancer*, **61**, 382-384.

GILLEN CD, WALMSLEY RS, PRIOR P, ANDREWS HA AND ALLAN RN. (1994). Ulcerative colitis and Crohn's disease: a comparison of the colorectal cancer risk in extensive colitis. *Gut*, **35**, 1590-1592.

JASS JR AND STEWART SM. (1992). Evolution of hereditary non-polyposis colorectal cancer. *Gut*, **33**, 783-786.

KINZLER KW, NILBERT MC, SU L-K, VOGELSTEIN B, BRYAN TM, LEVY DB, SMITH KJ, PREISINGER AC, HEDGE P, McKECHNIE D, FINNIEAR R, MARKHAM A, GROFFEN J, BOGUSKI MS, ALTSCHUL SF, HORII A, ANDO H, MIYOSHI Y, MIKI Y, NISHISHO I, NAKAMURA Y. (1991). Identification of FAP locus genes from chromosome 5q21. *Science*, **253**, 661-665.

LEACH FS, NICOLAIDES NC, PAPADOPOULOS N, LIU B, JEN J, PARSONS R, PELTOMÄKI P, SISTONEN P, AALTONEN LA, NYSTRÖM-LAHTI M, GUAN X-Y, ZHANG J, MELTZER PS, YU J-W, KAO F-T, CHEN DJ, CEROSALETTI KM, FOURNIER REK, TODD S, LEWIS T, LEACH RJ, NAYLOR SL, WEISSENBACH J, MECKLIN J-P, JÄRVINEN H, PETERSEN GM, HAMILTON SR, GREEN J, JASS J, WATSON P, LYNCH HT, TRENT JM, DE LA CHAPELLE A, KINZLER KW AND VOGELSTEIN B. (1993). Mutations of a *mutS* homolog in hereditary nonpolyposis colorectal cancer. *Cell*, **75**, 1215-1225.

LINDBLOM A, TANNERGÅRD P, WERELIUS B AND NORDENSKJÖLD M. (1993). Genetic mapping of a second locus predisposing to hereditary non-polyposis colon cancer. *Nature Genet.*, **5**, 279-282.

LYNCH HT, SMYRK TC, WATSON P, LANSPA SJ, LYNCH JF, LYNCH PM, CAVALIERI RJ AND BOLAND CR. (1993). Genetics, natural history, tumour spectrum, and pathology of hereditary nonpolyposis colorectal cancer: an updated review. *Gastroenterology*, **104**, 1535-1549.

NICOLAIDES NC, PAPADOPOULOS N, LIU B, WEI Y-F, CARTER KC, RUBEN SM, ROSEN CA, HASELTINE WA, FLEISCHMANN RD, FRASER CM, ADAMS MD, VENTER JC, DUNLOP MG, HAMILTON SR, PETERSEN GM, DE LA CHAPELLE A, VOGELSTEIN B AND KINZLER KW. (1994). Mutations of two *PMS* homologues in hereditary nonpolyposis colon cancer. *Nature*, **371**, 75-80.

PARSONS R, LI G-M, LONGLEY MJ, FANG W, PAPADOPOULOS N, JEN J, DE LA CHAPELLE A, KINZLER KW, VOGELSTEIN B AND MODRICH P. (1993). Hypermutability and mismatch repair deficiency in RER$^+$ tumour cells. *Cell*, **75**, 1227-1236.

PELTOMÄKI P, AALTONEN LA, SISTONEN P, PYLKKÄNEN L, MECKLIN J-P, JÄRVINEN H, GREEN JS, JASS JR, WEBER JL, LEACH FS, PETERSEN GM, HAMILTON SR, DE LA CHAPELLE A AND VOGELSTEIN B. (1993). Genetic mapping of a locus predisposing to human colorectal cancer. *Science*, **260**, 810-812.

PHILLIPS RKS, SPIGELMAN AD AND THOMPSON JPS. (eds). (1994). *Familial Adenomatous Polyposis and Other Polyposis Syndromes*. Edward Arnold: London.

SU L-K, VOGELSTEIN B, KINZLER KW. (1993). Association of the APC tumour suppressor protein with catenins. *Science*, **262**, 1734-1737.

VASEN HFA, MECKLIN J-P, MEERA KHAN P, LYNCH HT. (1991). The international collaborative group on hereditary nonpolyposis colorectal cancer. *Dis. Colon Rect.*, **34**, 424-425.

VEH RW, MEESSEN D, KUNTZ HD AND MAY B. (1982). A new method for histochemical demonstration of side chain substituted sialic acids. In *Colonic Carcinogenesis*, Malt RA and Williamson RCN. (eds) pp. 335-365. MTP Press: Lancaster.

VOGEL F AND MOTULSKY AG. (1986). *Human Genetics: Problems and Approaches*, 2nd edn, pp. 129-130. Springer: Berlin.


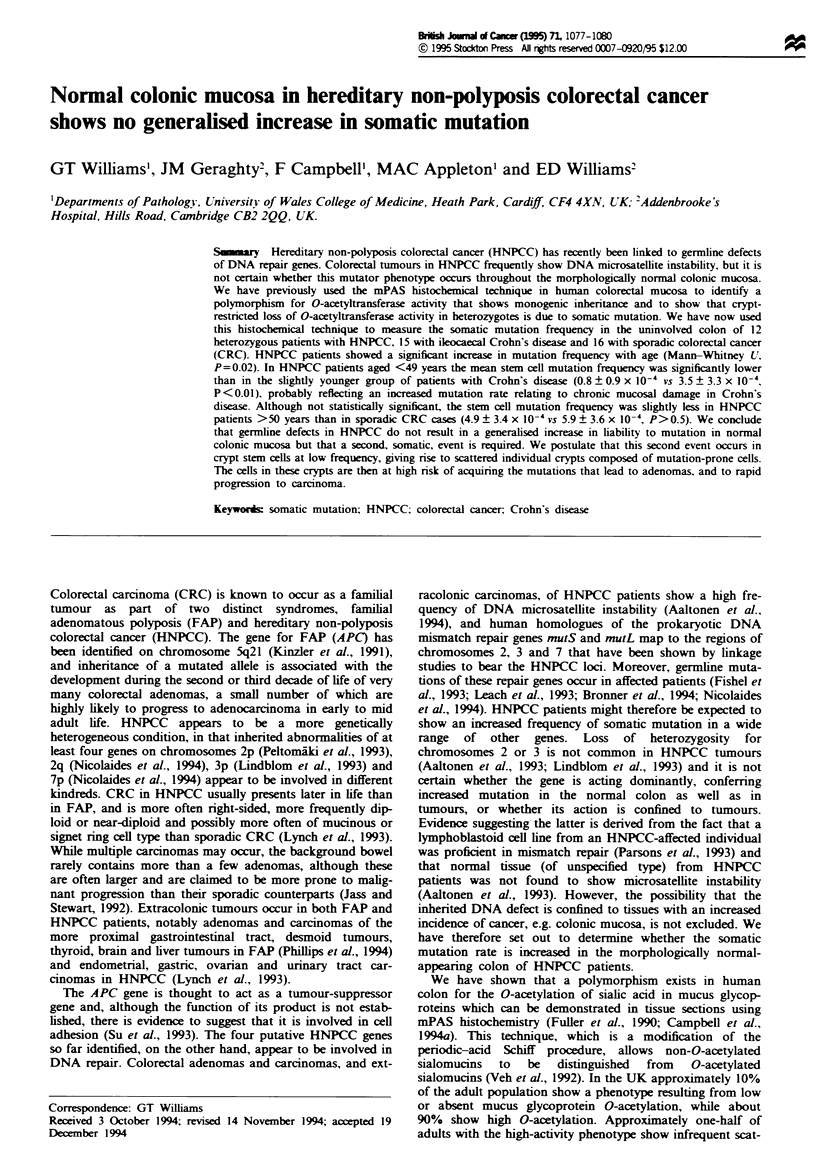

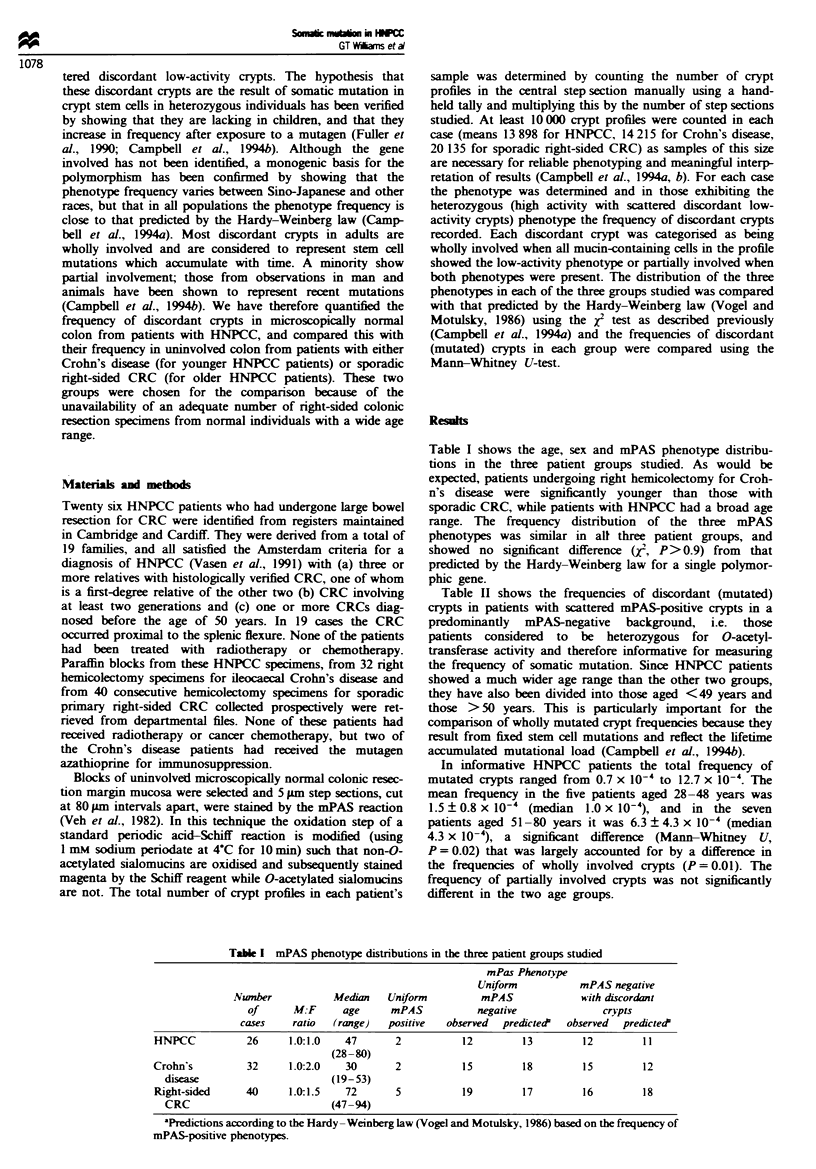

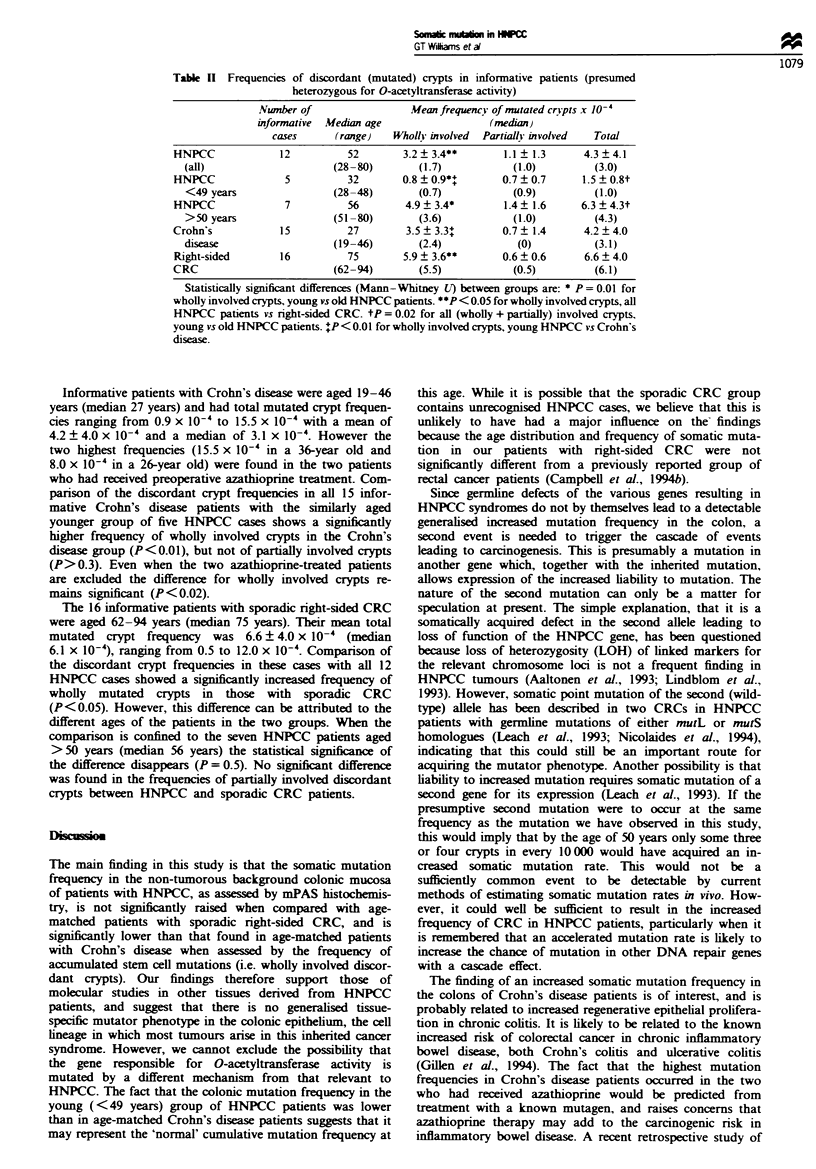

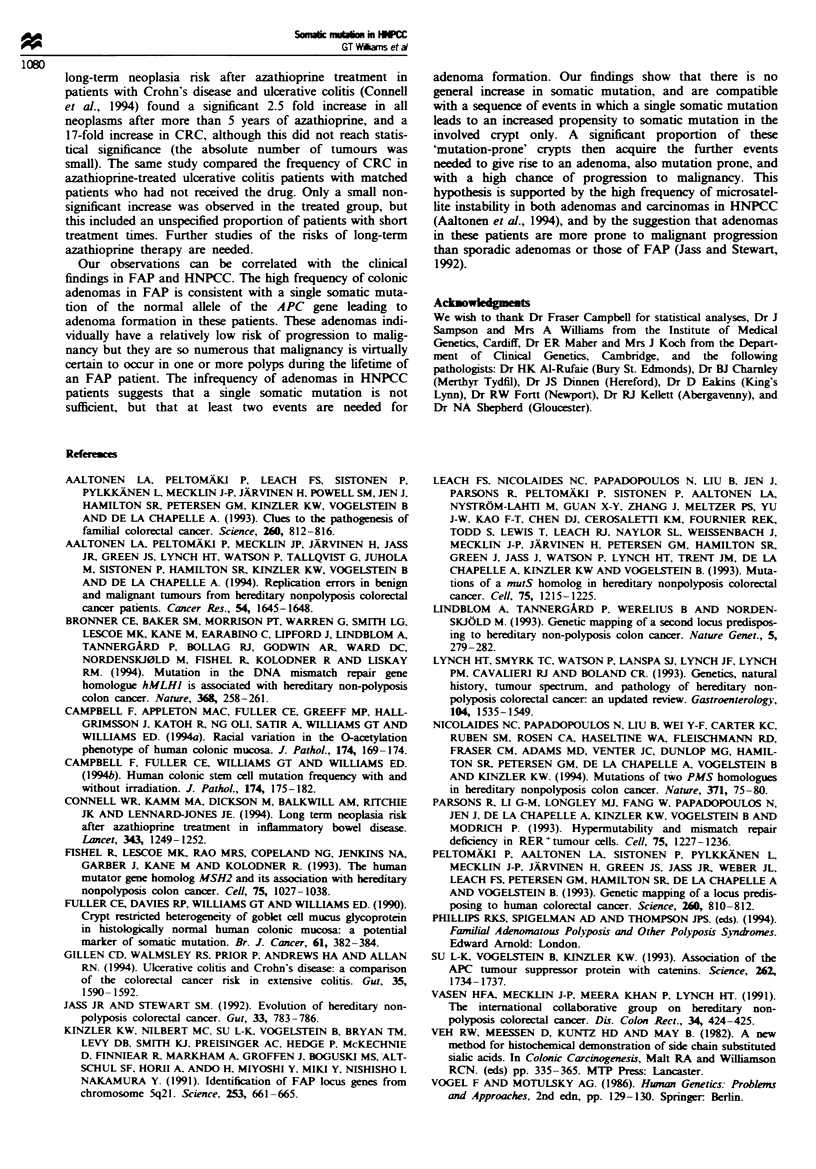


## References

[OCR_00480] Aaltonen L. A., Peltomäki P., Leach F. S., Sistonen P., Pylkkänen L., Mecklin J. P., Järvinen H., Powell S. M., Jen J., Hamilton S. R. (1993). Clues to the pathogenesis of familial colorectal cancer.. Science.

[OCR_00487] Aaltonen L. A., Peltomäki P., Mecklin J. P., Järvinen H., Jass J. R., Green J. S., Lynch H. T., Watson P., Tallqvist G., Juhola M. (1994). Replication errors in benign and malignant tumors from hereditary nonpolyposis colorectal cancer patients.. Cancer Res.

[OCR_00501] Campbell F., Appleton M. A., Fuller C. E., Greeff M. P., Hallgrimsson J., Katoh R., Ng O. L., Satir A., Williams G. T., Williams E. D. (1994). Racial variation in the O-acetylation phenotype of human colonic mucosa.. J Pathol.

[OCR_00508] Campbell F., Fuller C. E., Williams G. T., Williams E. D. (1994). Human colonic stem cell mutation frequency with and without irradiation.. J Pathol.

[OCR_00513] Connell W. R., Kamm M. A., Dickson M., Balkwill A. M., Ritchie J. K., Lennard-Jones J. E. (1994). Long-term neoplasia risk after azathioprine treatment in inflammatory bowel disease.. Lancet.

[OCR_00519] Fishel R., Lescoe M. K., Rao M. R., Copeland N. G., Jenkins N. A., Garber J., Kane M., Kolodner R. (1993). The human mutator gene homolog MSH2 and its association with hereditary nonpolyposis colon cancer.. Cell.

[OCR_00523] Fuller C. E., Davies R. P., Williams G. T., Williams E. D. (1990). Crypt restricted heterogeneity of goblet cell mucus glycoprotein in histologically normal human colonic mucosa: a potential marker of somatic mutation.. Br J Cancer.

[OCR_00531] Gillen C. D., Walmsley R. S., Prior P., Andrews H. A., Allan R. N. (1994). Ulcerative colitis and Crohn's disease: a comparison of the colorectal cancer risk in extensive colitis.. Gut.

[OCR_00537] Jass J. R., Stewart S. M. (1992). Evolution of hereditary non-polyposis colorectal cancer.. Gut.

[OCR_00561] Lindblom A., Tannergård P., Werelius B., Nordenskjöld M. (1993). Genetic mapping of a second locus predisposing to hereditary non-polyposis colon cancer.. Nat Genet.

[OCR_00568] Lynch H. T., Smyrk T. C., Watson P., Lanspa S. J., Lynch J. F., Lynch P. M., Cavalieri R. J., Boland C. R. (1993). Genetics, natural history, tumor spectrum, and pathology of hereditary nonpolyposis colorectal cancer: an updated review.. Gastroenterology.

[OCR_00572] Nicolaides N. C., Papadopoulos N., Liu B., Wei Y. F., Carter K. C., Ruben S. M., Rosen C. A., Haseltine W. A., Fleischmann R. D., Fraser C. M. (1994). Mutations of two PMS homologues in hereditary nonpolyposis colon cancer.. Nature.

[OCR_00543] Nishisho I., Nakamura Y., Miyoshi Y., Miki Y., Ando H., Horii A., Koyama K., Utsunomiya J., Baba S., Hedge P. (1991). Mutations of chromosome 5q21 genes in FAP and colorectal cancer patients.. Science.

[OCR_00577] Parsons R., Li G. M., Longley M. J., Fang W. H., Papadopoulos N., Jen J., de la Chapelle A., Kinzler K. W., Vogelstein B., Modrich P. (1993). Hypermutability and mismatch repair deficiency in RER+ tumor cells.. Cell.

[OCR_00589] Peltomäki P., Aaltonen L. A., Sistonen P., Pylkkänen L., Mecklin J. P., Järvinen H., Green J. S., Jass J. R., Weber J. L., Leach F. S. (1993). Genetic mapping of a locus predisposing to human colorectal cancer.. Science.

[OCR_00598] Su L. K., Vogelstein B., Kinzler K. W. (1993). Association of the APC tumor suppressor protein with catenins.. Science.

[OCR_00603] Vasen H. F., Mecklin J. P., Khan P. M., Lynch H. T. (1991). The International Collaborative Group on Hereditary Non-Polyposis Colorectal Cancer (ICG-HNPCC).. Dis Colon Rectum.

